# Faculty Development – Full Steam Ahead!

**DOI:** 10.3205/zma001127

**Published:** 2017-10-16

**Authors:** Götz Fabry, Anja Härtl

**Affiliations:** 1 Albert-Ludwig-Universität Freiburg, Abt. für Med. Psychologie, Freiburg/Brg, Germany; 2 GMS Journal for Medical Education, Assistant Chief Editor, Erlangen, Germany; 3 Klinikum der LMU München, Institut für Didaktik und Ausbildungsforschung in der Medizin, München, Germany

## Editorial

Almost 15 years ago, we were facing the challenge to find a name for the GMA committee working on issues that in the Anglo-American language world are concisely called “faculty development”. The obvious German term “Fakultätsentwicklung” would not have been very accurate as “faculty” especially in North America denotes the personnel rather than the institution (whereas the German term “Fakultät” almost exclusively denotes the institution). To include both aspects even-handedly – individuals as well as the institution – we decided after some discussion to use the bulky but more precise term “Personal- und Organisationsentwicklung” [personal and organizational development] which is also used in other professional fields. We supplemented it with “in der Lehre” [in education] to distinguish it from other aspects of personal and organizational development. As the articles in this themed issue illustrate this was a good choice because the spectrum of initiatives, projects, and scientific activities in the field grew enormously since then. Initially, the main focus was the development and implementation of faculty development initiatives at the medical schools for qualifying the faculty comprehensively to fulfill their tasks in medical education [1]. Meanwhile, all medical schools offer some faculty development for higher or medical education respectively. Furthermore, as a result of the work within the MedizinDidaktikNetz Deutschland, a network of all medial schools dedicated to faculty development [https://goo.gl/WrGFYJ], a common standard for qualification steps in faculty development was achieved. It is much easier now for medical teachers to qualify themselves appropriately and to improve the quality of their teaching. Not only courses for basic knowledge in higher and medical education are offered but also workshops and trainings for more specific tasks in medical education as two articles in this issue demonstrate: Henrike Hölzer et al. report on a training to facilitate small groups with simulated patients [2] and Konstanze Vogt et al. describe a teacher training for problem-based learning [3].

In recent years, a documented qualification for teaching has also become more important regarding academic promotion and tenure, e.g. as a prerequisite for the “Habilitation” (a necessary requirement to apply for a full professorship in medicine). Although the individual teaching merits are still not rated as equally important for promotion and tenure as the individual academic achievements, there is growing awareness that research and teaching should be treated as equal. Furthermore, it is widely accepted now that a qualification for teaching in higher education does not simply appear from nowhere. As Marianne Merkt states in her comment, there is still a lot of room for improvement in this regard and preliminary work from other countries we can build on [4].

Teaching in higher education is increasingly in the public eye and a topic of political debate. In 2011 for instance, the “Qualitätspakt Lehre” [https://goo.gl/CsdjxS], a political initiative launched by the federal ministry of education and research to promote the quality of teaching and learning in higher education, generated many projects at German universities. The dynamic development regarding teaching also sparked a more fundamental debate about what it means to be a good educator in higher education and what we mean by “good education” [5]. A central aspect of this discussion is the request to understand teaching and learning as a scholarly activity (“scholarship of teaching and learning”) and to design the curricula respectively e.g. by research-based teaching [6]. In addition, against the background of the general discussion regarding competency based education in all parts of the educational system, frameworks have been developed to also define competencies for teaching personnel in higher education [7], [8]. On the one hand, these frameworks are important to enhance faculty development initiatives in higher and medical education and Jan Griewatz et al. report an example of a well-established teacher training that was revised to make it more competency-based [9]. The concept of the Waldbreitbacher Ärzteakademie, an institution for postgraduate education, reported by Katrin Keller et al., is another example [10] in this regard.

On the other hand, these frameworks also document that competencies for higher and medical education are more than just knowledge regarding different teaching methods and that a comprehensive training on all levels of the educational continuum is needed to master them. As Niclas Schaper points out in his comment published in this issue, it is important that these frameworks are not only evaluated regarding their usefulness, but also to check their validity i.e. to analyze the underlying constructs [11]. Anike Hertel-Waszak et al. give an example how such an evaluation might be carried out: physicians, other health professionals and patients were asked to define core competencies for physicians [12]. The results show a high degree of agreement with the National Catalogue for Competency-based Learning Objectives in Medicine (NKLM) which might be taken as a piece of evidence for the validity of this document.

These kind of validity studies are examples of research in faculty development. However, hitherto the focus of research is primarily on the evaluation of the acceptance and the benefit of training initiatives in higher and medical education [13]. To this end, the participants of these trainings are often surveyed regarding their satisfaction and self-assessed growth in competencies. Given the multitude of variables that influence the transfer to the workplace, the validity of these results is limited yet. The article by Julia Freytag et al. is an exception in this regard, as the success of a course for working with simulated patients was assessed by direct observation while the participants were teaching [14].

As Yvonne Steinert states in her comment though, it is important to supplement the research paradigms and methods used so far to move beyond this kind of evidence and to understand better why and how faculty development for teaching works [15]. As becomes apparent in educational research in general, qualitative methods are especially promising in this regard. Given the high amount of complexity in the learning environments in which we as teachers work and the resulting multitude of variables that influence the quality of teaching and learning for instance, quantitative studies are very challenging or limited in their significance. It is the strength of qualitative studies or mixed-method-designs to capture these complex processes prototypically and to discover patterns or important variables. But it is not only important to broaden our methodological repertoire; the spectrum of the questions we inquire should also be widened. Along the lines of the development in undergraduate and postgraduate medical education, it is also worthwhile in faculty development to focus more on the significance of implicit learning at the workplace and in communities of practice. It is especially important here to understand how skills and competencies that have been learned in respective trainings transfer into daily practice. Beyond the questions that are specifically interesting from a medical education point of view it is also worthwhile and relevant to consider problems that affect teachers in higher education generally and thus, to also turn to the literature outside the realm of medical education.

In addition to faculty development in terms of workshops, courses or trainings, there are environmental variables at a medical school or other institutions for undergraduate or postgraduate medical training that influence teaching and learning and thus, the quality of teaching and its advancement. Marianne Giesler et al. attempt to arrange the multitude of these variables by means of the “Frankfurt Model” which is published here for the first time [16]. They also provide a checklist to tackle the variables pragmatically. Two examples of how these variables might foster or hinder faculty development are also published in this issue: Ulrike Sonntag et al. report on the experiences with a course for basic educational competencies at the Charité [17] and Thomas Kollewe et al. on the experiences with the implementation of an office for medical education in Frankfurt [18]. As both contributions cover the timespan of a decade, they also relate to long-term developments.

Overall, this themed issue clearly shows that the field of faculty development in the German speaking countries develops dynamically and bears a large potential for research as well as for the practical work at the medial schools. However, it also becomes apparent that some themes are not discussed (yet), which might be the object of future activities. In light of the academization of health professions such as nursing or physiotherapy it is an interesting question for instance, how teachers in these fields, who often have a professional teacher training already, continue their professional educational development. Since interprofessional education will be more and more integrated in the medical undergraduate as well as postgraduate curricula we also need respective faculty development initiatives for the teachers who are expected to implement these concepts [19]. Another challenge for teaching and learning in many places is the digitalization and there seems to be no consensus yet which competencies teachers need to master this challenge in a sound pedagogical way. New assessment formats and the development towards “programmatic assessment” also require new competencies from examiners as well as learners [20]. Other disciplines such as dental or veterinary medicine do also revise and advance their curricula and qualify their teachers but these initiatives have hardly been published hitherto in the German language area. Aside from the individuals, it is also an important question how the organizations can further meet the demands placed upon them. What kind of organizational structures and conditions would be suitable to develop a school that fosters international, intercultural, interprofessional and competency-based teaching and learning? Individuals who organize, plan, and manage teaching and constitute the so-called “third space” have rarely been the subject of faculty development for teaching so far [21]. At the same time, these individuals are indispensable for the advancement of the medical schools, because without them no assessments, no teaching or faculty development etc. would take place. Aside from formal faculty development, the informal ways to learn are increasingly brought into the focus of research. An important question in this regard is how the professional identity of an individual in the health professions develops. Again, studies to inquire this have rarely been undertaken in the German language area up to now. All these question are not only relevant for teachers in undergraduate medical education, but also for teachers in postgraduate medical training.

These examples for themes that might be discussed in the future within faculty development in teaching raise hope that the field will continue to develop and grow dynamically and excitingly.

## Competing interests

The authors declare that they have no competing interests. 
